# Mortality of Chicago, for 1856

**Published:** 1857-02

**Authors:** 


*Mortality of Chicago, for* 1856.

According to the City Sexton's records, the whole number of deaths in this city during the year 1856, was 1950; of which 550 were under *five* years of age, 144 between five and ten, 62

between ten and twenty, 273 between twenty and thirty, 133 between thirty and forty, 51 between forty and fifty, 27 between fifty and sixty, 19 between sixty and seventy, 14 between seventy and eighty, and 2 over eighty years of age. If we allow the city an average population during the year of 95,000, which is probably considerably below the actual number, the ratio of mortality for the year would be only 1 in 49 of the inhabitants. This is a lower ratio than the average for Philadelphia, or any of the cities along the Atlantic coast.

